# Efficacy and safety of Shugan Jieyu capsule in the treatment of essential hypertension with insomnia, anxiety or depression: A protocol for systematic review and meta-analysis

**DOI:** 10.1097/MD.0000000000024856

**Published:** 2021-02-26

**Authors:** Maoxia Fan, Dong Guo, Ying Tian, Yongcheng Liu, Jisen Zhao

**Affiliations:** a The First Clinical Medical College of Shandong University of Traditional Chinese Medicine; b Teacher Development Center of Shandong University of Traditional Chinese Medicine; c Shandong Provincial Hospital of Traditional Chinese Medicine, Jinan, Shandong Province, China.

**Keywords:** anxiety, depression, essential hypertension, insomnia, regimen, Shugan Jieyu capsule, systematic review

## Abstract

**Background:**

Shugan Jieyu capsule can reduce blood pressure and improve its concomitant symptoms. However, it is not widely used in clinic because of its incomplete understanding of its nature. There are many reports on the clinical trials of Shugan Jieyu capsule in the treatment of essential hypertension with insomnia, anxiety or depression in recent years. However, the lack of systematic review and meta-analysis has not provided effective evidence. As a consequence, we provide a protocol to evaluate the efficacy and safety of Shugan Jieyu capsule (SJC) in the treatment of essential hypertension (EH) with insomnia, anxiety or depression.

**Methods:**

The search time range of Cochrane Library, PubMed, excerpt Database (EMBASE), Chinese Biomedical Literature Database (CBM), China National knowledge Infrastructure (CNKI), Chinese Science and Technology Journal Database (VIP), and Wanfang Database (WanFang), was searched by computer from the establishment of the database to December 31, 2020. In the meanwhile, the list of references and related reviews were checked. The data were extracted by 2 evaluators independently, and the literature quality was evaluated according to Cochrane manual 4.2.2. In addition, CochraneRevman5.3 software was used for heterogeneity test, meta-analysis, publication bias analysis and GRADE3.6 evidence quality classification system evaluation related statistical data.

**Results:**

This study intends to evaluate the efficacy and safety of SJC in the treatment of EH from many aspects, including changes in blood pressure [systolic blood pressure (SBP), diastolic blood pressure (DBP)], effective rate of blood pressure reduction, improvement rate of concomitant symptoms and adverse reactions.

**Conclusion:**

The conclusion of systematic review intends to provide evidence for judging that SJC is an effective intervention for EH patients with insomnia, anxiety and depression.

**PROSPERO registration number:**

PROSPERO CRD 42021219704.

## 1 Introduction

As one of the risk factors of cardiovascular and cerebrovascular diseases, essential hypertension (EH) can lead to the damage of heart, brain, kidney and other target organs, and the disability rate and mortality rate are very high.^[1,2]^ The prevalence rate of hypertension in China has increased rapidly from 9.8% in 1980 s to more than 30% in the 21st century with the development of economy and society.^[3]^ There are currently 245 million patients with essential hypertension in China according to the data released by the 2018 China Cardiovascular Disease report.^[4]^ The survey shows that the prevalence rate of EH in China is still on the rise, and the control rate is at a low level,^[5]^ which has become an important public health problem. EH is a typical psychosomatic disease, resulting in more and more pressure in people's life, work, family and many other aspects with the acceleration of the pace of modern life. The incidence of hypertension complicated with insomnia, anxiety, depression and other physical and psychological diseases is increasing. Anxiety and depression have a two-way relationship with chronic diseases such as EH.^[6,7]^ Anxiety and depression form chronic stimulation to the sympathetic nervous system, which leads to insulin resistance and affects the function of the heart and blood vessels.^[6]^

The modern pharmacological study of hypericin in Shugan Jieyu capsule (SJC) proved its antidepressant effect. Acanthopanax senticosus can resist fatigue, sedation, regulate cellular immunity and humoral immunity, etc.^[8]^ In addition, SJC can also increase the secretion of dopamine and 5-HT to improve nerve excitability and play the role of anti-anxiety and depression.^[9]^ The SJC made by the combination of Hypericum perforatum and Acanthopanax senticosus can effectively relieve the liver and relieve depression, calm the mind, replenish qi and invigorate the spleen,. In other words, it can reduce the patient's blood pressure, improve the patient's anxiety, depression and other bad emotions. In the meanwhile, Acanthopanax senticosus can improve the patient's self-feeling, produce anti-fatigue, anti-stress, and enhance the patient's attention by regulating the central nervous system and immune system, protecting neurons, etc. It is more beneficial for patients to enter the sleep state.^[10]^

There are many reports on clinical trials of SJC combined with conventional antihypertensive drugs in the treatment of EH with insomnia, anxiety or depression in recent years. However, there is a lack of systematic review to evaluate the clinical efficacy. As a consequence, this protocol is prepared for systematic review and meta-analysis to evaluate its effectiveness and safety and to provide more high-quality evidence for clinical drug use.

## 2 Methods

### 2.1 Inclusion criteria for study selection

#### 2.1.1 Research type

All published and unpublished randomized and semi-randomized controlled trials; languages are not limited.

#### 2.1.2 Patient type

The subjects were in line with the international and domestic recognized diagnostic criteria for hypertension; the patients included in the trial were hypertension patients with insomnia, anxiety or depression, or 2 or 3 of them were accompanied.

#### 2.1.3 Intervention measures

The experimental group was treated with SJC+routine antihypertensive drugs, and the control group was treated with conventional antihypertensive drugs or conventional antihypertensive drugs+placebo.

#### 2.1.4 Outcome index

The criteria for judging the expected outcome were clear, including at least the changes of blood pressure [systolic Blood pressure (SBP), diastolic Blood pressure (DBP)], the effective rate of reducing blood pressure (including significant effect, effective, ineffective, etc.), the improvement rate of concomitant symptoms (PSQI scale score, anxiety scale score, depression scale score), and adverse reactions.

#### 2.1.5 Electronic retrieval

The computer searches the relevant journals of Cochrane Library, PubMed, excerpt Database (EMBASE), Chinese Biomedical Literature Database (CBM), China National knowledge Infrastructure (CNKI), Chinese Science and Technology Journal Database (VIP), and Wanfang Database (WanFang) according to the retrieval strategy, and the retrieval time ranges from the establishment of the database to July 30, 2020. References that meet the inclusion criteria of the study were reviewed one by one to avoid omissions. SJC and essential hypertension and their synonyms were search terms. Search strategy for the PubMed database (Table 1).

**Table 1:** Search strategy for PubMed.

Number	Search terms
1	#1 Search (“Essential Hypertension” [Mesh]) OR (“Hypertension” [Mesh]) OR (“Blood Pressure, High” [Mesh]) OR (“Blood Pressures, High” [Mesh]) OR (“High Blood Pressure” [Mesh]) OR (“High Blood Pressures” [Mesh])
2	#2 Search (“Shugan Jieyu capsules”) OR (“Shugan Jieyu capsules”)
3	#3 Search (“randomized controlled trial” [Title/Abstract]) OR (“randomized” [Title/Abstract]) OR (“randomly” [Title/Abstract])
4	#1 AND #2 AND #3

### 2.2 Data collection and analysis

#### 2.2.1 Research and appraisal

**Figure 1:**
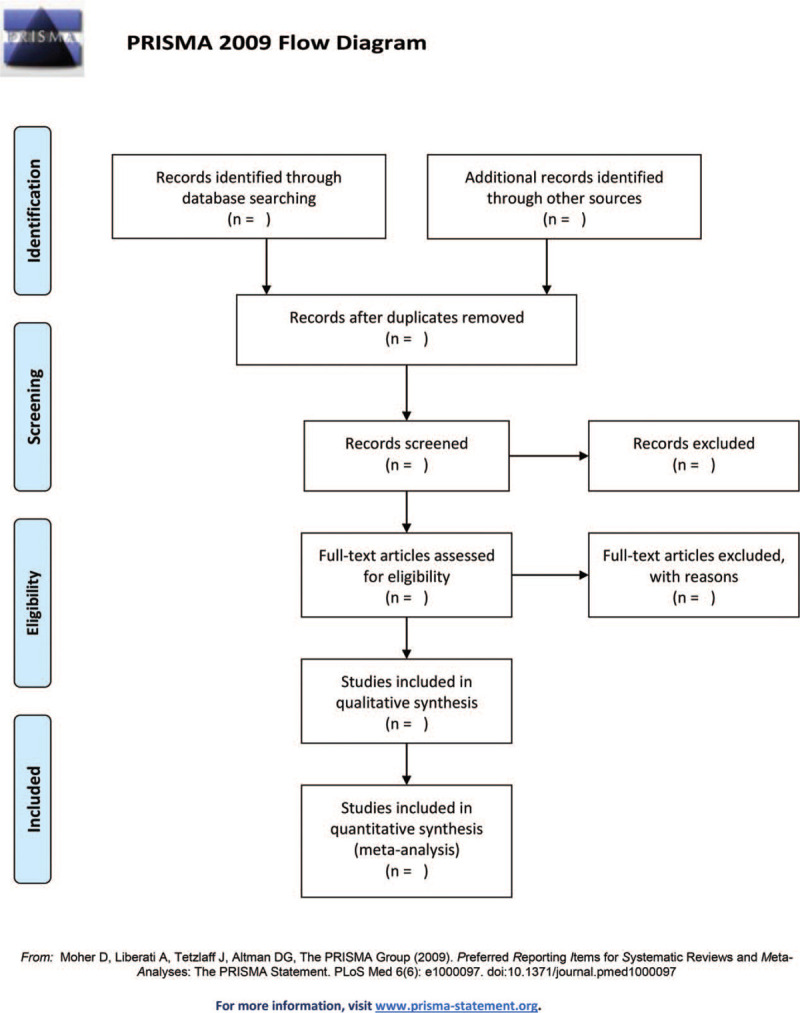
PRISMA 2009 Flow Diagram.

Two researchers conducted independent and cross-check literature screening. They should discuss with each other to propose a solution or consult another research member if they disagree. If necessary, contact the original research author by email or telephone for uncertain but important information. First read the title of the reference to exclude the apparently irrelevant literature. In addition, read the abstract and the full text to determine whether these studies are finally included. The process of research selection is given in the (PRISMA) flowchart of “preferred reporting items for system review and meta-analysis” (Fig. 1).

#### 2.2.2 Extraction result

The 2 evaluators independently extracted the data according to the predesigned extraction form, which included: (a) basic situation: study number, patient age, sex, study design, number of trials in each group, baseline comparability; (b) intervention measures: different intervention measures and courses of treatment between the test group and the control group; (c) the elements of risk bias assessment; (d) the results of the study: the outcome indicators needed for this systematic evaluation.

#### 2.2.3 Quality evaluation of literature methodology

The methodology of the included literature was evaluated according to Cochrane manual 5.01. Evaluation criteria included random methods, random sequence generation, random hiding, blind patients and researchers, blind outcome evaluators, incomplete outcome reports, and selective outcome reports. It were evaluated independently by 2 evaluators according to the unified quality standard, which was divided into 3 levels: “low,” “unclear,” or, “high”. The 2 sides discussed the solution or consulted the third evaluator if there was any disagreement.

#### 2.2.4 Heterogeneity evaluation

The Revman5.3 software provided by Cochrane website was used for analysis. The feasibility of meta-analysis of heterogeneity was assessed between trials. If the *I*^2^ value is more than 50%, we intend to consider the existence of significant heterogeneity and conduct a subgroup analysis to investigate the potential causes of clinical or methodological heterogeneity.

#### 2.2.5 Publication bias

If there were at least 10 experimental studies, the visual asymmetry on the funnel chart was used to determine whether there was a publication bias.

#### 2.2.6 Data analysis

The Revman5.3 software provided by Cochrane website was used for analysis. The classified variables were expressed by the odds ratio (OR), and the 95% confidence interval (CI), continuous variables were expressed by the standard mean difference (SMD). The heterogeneity of each included study was tested, when *P* > .1 or *I*^2^ < 50%, the heterogeneity among the groups was small, and the fixed effect model was used for combined analysis; when *P* < .1 or *I*^2^ > 50%, the heterogeneity among the groups was large, then the random effect model was used for combined analysis, and the results were shown by forest map. Sensitivity analysis and subgroup analysis were used if necessary. α = 0.05 was considered to be statistically significant. If meta-analysis was not possible, we only described the data.

#### 2.2.7 Subgroup analysis

If there were at least 10 trials included, a subgroup analysis would be conducted based on different interventions, participants, gender, treatment duration, and drug dose to explore the source of heterogeneity.

#### 2.2.8 Sensitivity analysis

Sensitivity analysis was conducted according to sample size, missing data and methodological quality to identify the quality.

#### 2.2.9 GRADE evidence quality rating^[11]^

The GRADE evidence quality evaluation system was used to evaluate the evidence quality for the results of this systematic review. The evidence quality was divided into 4 levels: high (- 0), medium (- 1), low (- 2), and very low (- 3), and the recommended level was divided into strong and weak levels.

## 3 Conclusion

This was a study to evaluate the clinical efficacy and safety of SJC in the treatment of insomnia, anxiety or depression in patients with EH. Traditional Chinese medicine has certain advantages in the comprehensive management of EH. However, cooperating with western medicine to reduce blood pressure steadily, it can better regulate vascular endothelial function, improve blood composition, protect tissues and organs,^[12]^ and improve concomitant symptoms at the same time.

SJC is a pure compound preparation of traditional Chinese medicine composed of Hypericum perforatum and Acanthopanax senticosus. Hypericum perforatum has the effects of soothing the liver and relieving depression, clearing heat and dampness, reducing swelling and pain.^[13]^ Acanthopanax senticosus has the effects of sedation, anti-fatigue, replenishing qi and invigorating spleen, tonifying kidney and tranquilizing mind.^[14]^ The 2 work together to reduce blood pressure and improve the curative effect of concomitant symptoms. We hope that this systematic review intends to provide more convincing evidence for SJC in the treatment of EH. However, this systematic review has some limitations: firstly, the heterogeneity will be increased due to the age, severity and treatment time of EH patients; secondly, small samples may lead to high-risk bias. As a consequence, randomized controlled trials (RCT) with higher quality and low bias risk are needed to further explore the effectiveness and safety of SJC in order to better guide clinical drug use in the future.

## Author contributions

**Conceptualization:** Maoxia Fan.

**Data curation:** Ying Tian, Yongcheng Liu.

**Formal analysis:** Maoxia Fan, Dong Guo, Jisen Zhao.

**Investigation:** Ying Tian, Yongcheng Liu.

**Methodology:** Maoxia Fan.

**Software:** Maoxia Fan, Jisen Zhao.

**Writing – original draft:** Maoxia Fan.

**Writing – review & editing:** Maoxia Fan, Dong Guo.
